# A “Candidate-Interactome” Aggregate Analysis of Genome-Wide Association Data in Multiple Sclerosis

**DOI:** 10.1371/journal.pone.0063300

**Published:** 2013-05-16

**Authors:** Rosella Mechelli, Renato Umeton, Claudia Policano, Viviana Annibali, Giulia Coarelli, Vito A. G. Ricigliano, Danila Vittori, Arianna Fornasiero, Maria Chiara Buscarinu, International Multiple Sclerosis Genetics Consortium, Wellcome Trust Case Control Consortium,2, Silvia Romano, Marco Salvetti, Giovanni Ristori

**Affiliations:** Centre for Experimental Neurological Therapies, S. Andrea Hospital-site, Department of Neuroscience, Mental Health and Sensory Organs (NESMOS), Faculty of Medicine and Psychology, Sapienza University, Rome, Italy; University of Nebraska - Lincoln, United States of America

## Abstract

Though difficult, the study of gene-environment interactions in multifactorial diseases is crucial for interpreting the relevance of non-heritable factors and prevents from overlooking genetic associations with small but measurable effects. We propose a “candidate interactome” (i.e. a group of genes whose products are known to physically interact with environmental factors that may be relevant for disease pathogenesis) analysis of genome-wide association data in multiple sclerosis. We looked for statistical enrichment of associations among interactomes that, at the current state of knowledge, may be representative of gene-environment interactions of potential, uncertain or unlikely relevance for multiple sclerosis pathogenesis: Epstein-Barr virus, human immunodeficiency virus, hepatitis B virus, hepatitis C virus, cytomegalovirus, HHV8-Kaposi sarcoma, H1N1-influenza, JC virus, human innate immunity interactome for type I interferon, autoimmune regulator, vitamin D receptor, aryl hydrocarbon receptor and a panel of proteins targeted by 70 innate immune-modulating viral open reading frames from 30 viral species. Interactomes were either obtained from the literature or were manually curated. The P values of all single nucleotide polymorphism mapping to a given interactome were obtained from the last genome-wide association study of the International Multiple Sclerosis Genetics Consortium & the Wellcome Trust Case Control Consortium, 2. The interaction between genotype and Epstein Barr virus emerges as relevant for multiple sclerosis etiology. However, in line with recent data on the coexistence of common and unique strategies used by viruses to perturb the human molecular system, also other viruses have a similar potential, though probably less relevant in epidemiological terms.

## Introduction

As in other multifactorial diseases, genome-wide association studies (GWAS) are providing important data about disease-associated loci in multiple sclerosis (MS) [1]. In parallel, sero-epidemiological studies are reinforcing the evidence that nonheritable factors such as Epstein-Barr virus (EBV) and vitamin D are associated with disease pathogenesis [2].

However, the effect size of the gene variants identified so far in MS appears small. It is therefore important (but difficult: Sawcer and Wason, 2012) [3] to establish if and in which cases (including those gene variants with small but measurable effect size that do not reach the significance threshold of GWAS) the interaction with nonheritable factors may help understand their true impact on disease pathogenesis [4]. Furthermore, as far as the sero-epidemiological associations are concerned, their causal relevance and underlying pathogenetic mechanisms become clearer if interpreted in the light of genetic data.

As an attempt to consider, beyond the statistical paradigms of GWAS analysis, which gene-environment interactions may associate with the development of MS, we performed an interrogation of GWAS data [1] through a “candidate interactome” approach, investigating statistical enrichment of associations in genes whose products “interact” with putative environmental risk factors in MS.

We elected to center the analysis on viral interactomes, based on the classical hypothesis of a viral etiology of MS. Importantly, we examined only direct interactions between viral and human proteins as it has recently been shown that these are the interactions that are more likely to be of primary importance for the phenotypic impact of a virus in “virally implicated diseases” [5]. The chosen interactomes reflect the compromise between informative size and potential relevance for MS. In detail, EBV was chosen as main association to be verified against phylogenetically related or unrelated viruses. Given the profound influence of EBV on the immune response, and the preponderance of (auto)immune-mediated mechanisms in the pathogenesis of the disease, we added two interactomes of immunological relevance, human innate immunity interactome for type I interferon (hu-IFN) and autoimmune regulator (AIRE). Finally, we included the vitamin D receptor (VDR) and the aryl hydrocarbon receptor (AHR) interactomes to evaluate, on the same grounds, also part of the molecular interactions that compose other established or emerging “environmental” associations.

## Methods

Seven interactomes were obtained from the literature: EBV [6], Human Immunodeficiency virus (HIV) [7], Hepatitis C virus (HCV) [8], AIRE [9], hu-IFN [10], Influenza A virus (H1N1) [11], Virus Open Reading Frame (VIRORF) [12]. Four interactomes were manually curated: Human Herpesvirus 8 (HHV8), Cytomegalovirus (CMV), JC virus (JCV), Hepatitis B virus (HBV). VDR and AHR interactomes were extracted from BIOGRID (http: //thebiogrid.org) [13].

As reference to gather gene and single nucleotide polymorphism (SNP) details from their HUGO Gene Nomenclature Committee (HGNC) Ids and rsids, we employed a local copy of the Ensembl Human databases (version 66, databases *core* and *variation*, including SNPs coming from the 1000 Genome project); the annotation adopted for the whole analysis was GRCh37-p6, that includes the release 6 patches (Genome Reference Consortium: human assembly data - GRCh37.p6 - Genome Assembly. http: //www.ncbi.nlm.nih.gov/genome/assembly/304538/).

The genotypic p-values of association for each tested SNP were obtained from the International Multiple Sclerosis Genetics Consortium & Wellcome Trust Case Control Consortium,2 study. All SNPs which did not pass quality checks in the International Multiple Sclerosis Genetics Consortium & the Wellcome Trust Case Control Consortium,2 study were filtered out from the original data. We used ALIGATOR [14], [15] to evaluate how single genes get summed to provide total contribution of candidate interactomes (Table S1). The idea behind ALIGATOR’s strategy is to evaluate gene category significance by means of an empirical approach, comparing each interactome with the null hypothesis, built using random permutations of the data. Such method begins its analyses by evaluating the Gene Ontology (GO) category association in each interactome provided: (i) each SNP with a p-value stronger than the P-CUT parameter is associated to the gene within 20 kb; then the most representative SNP for each gene is selected; (ii) LD filter of SNPs that have an r2≤0.2 and those that are farther than 1000 kb; (iii) count the number of genes significant in each GO category. This is the real observed data.

A non parametric bootstrap approach was used to generate a null hypothesis as follows: (i) build 5000 random interactomes (of the same size of the one under analysis, this procedure is repeated for each interactome); (ii) obtain category-specific p-values by comparing each random interactome with the remaining 4999 built; (iii) elect one of the interactomes in (i) as simulated observed data; (iv) randomly sample interactomes in (i) to generate category-specific p-values; (v) repeat (iv) to simulate 1000 simulated studies. The GO category association distribution in the real observed data is then compared with the null hypothesis: (i) generate an expected number of significant genes in each category, using the simulated studies; (ii) compare the number of significant categories in the real observed data with (i). ALIGATOR parameters that we used are those of its reference paper [14]. p-value cut-off was taken at 0.05, only the SNPs with marginal p-value less than this cut-off were employed (p-value cut-offs were also taken at 0.005 and 0.03 for the re-analysis of interactomes that resulted associated at 0.05, see results). Furthermore, to limit the uncertainties introduced by combined SNP effects in the MHC extended region (that is the haplotype set with the strongest signal in our analysis), we computed two different statistical evaluations for each interactome, one including and the other one excluding SNPs coming from such region (we considered as belonging to the extended MHC region all those SNPs that participate in at least one of the following haplotypes: HSCHR6_MHC_APD, HSCHR6_MHC_COX, HSCHR6_MHC_DBB, HSCHR6_MHC_MANN, HSCHR6_MHC_MCF, HSCHR6_MHC_QBL, HSCHR6_MHC_SSTO according to GRC data). In both cases we used Ensembl API [16] and BioPerl [17] (version 1.2.3) to gather all SNP information, haplotype participation, genes position and size [18]; such annotated information was then fed into ALIGATOR together with the interactomes.

Ingenuity Pathway Analysis (IPA) was employed twice: (i) before the ALIGATOR statistics, to characterize the composition of our interactomes (Table S2), and (ii) on the genes with nominally significant evidence of association [1] that ALIGATOR took as representative of each interactome-SNP relation (Table S3). In both cases we performed the IPA-”core analysis”, and we restricted the settings to show only molecular and functional associations. Afterwards, we used IPA-”comparative analysis” to produce the p-value of association between each functional class and all our interactomes. IPA knowledge base (ie, the input data used by IPA) was set to the following criteria in every analyses: consider only molecules and/or relationships where the species in object was human (or it was a chemical), and the datum was experimentally observed. Since IPA-”comparative analysis” provides p-value ranges associated to functional classes, we took as reference the value used by IPA to fill its reports, namely the best p-value for that class.

## Results

We performed a “candidate interactome” (i.e. a group of genes whose products are known to physically interact with environmental factors that may be relevant for disease pathogenesis) analysis of genome-wide association data in multiple sclerosis.

We obtained 13 interactomes, 7 from the literature (as such) and 6 by manually selecting those interactions that were reported by two independent sources or were confirmed by the same source with distinct experimental approaches. In all cases we considered only physical-direct interactions (Table S2,Table 1).

**Table 1:** Statistical enrichment of MS-associated genes within each interactome.

Interactome	Size	Source	p-value with MHC	p-value without MHC
VIRORF	579	Experimental data [12]	0.0610	0.0632
HIV	446	Experimental data [7]	0.0026	0.0034
HCV	202	Experimental data [8]	0.4244	0.4424
hu-IFN	113	Experimental data [10]	0.2176	0.1838
EBV	110	Experimental data [6]	0.0140	0.0446
H1N1	87	Experimental data [11]	0.9572	0.9648
AIRE	45	Experimental data [9]	0.4322	0.4012
HBV	85	manually curated	0.0124	0.0236
CMV	41	manually curated	0.1156	0.3322
HHV8	40	manually curated	0.1132	0.0920
JCV	10	manually curated	1.0000	1.0000
VDR	78	BioGRID	0.1848	0.1802
AHR	30	BioGRID	0.8752	0.8522

ALIGATOR-obtained interactome p-values (overall contribution given by SNP p-values to each interactome, with and without SNPs falling in the MHC region). The SNPs with marginal p-value less than 0.05 were employed.

MS = multiple sclerosis; ALIGATOR = Association LIst Go AnnoTatOR; SNP = single nucleotide polymorphism; MHC = Major histocompatibility complex; BioGRID = Biological General Repository for Interaction Datasets; VIRORF = Virus Open Reading Frame; HIV = Human Immunodeficiency virus; HCV = Hepatitis C virus; hu-IFN = human innate immunity interactome for type I interferon; EBV = Epstein Barr virus; H1N1 = Influenza A virus; HBV = Hepatitis B virus; VDR = vitamin D receptor; AIRE = autoimmune regulator; CMV = Cytomegalovirus; HHV8 = Human Herpesvirus 8; JCV = JC virus; AHR = Aryl hydrocarbon receptor.

**Figure 1:**
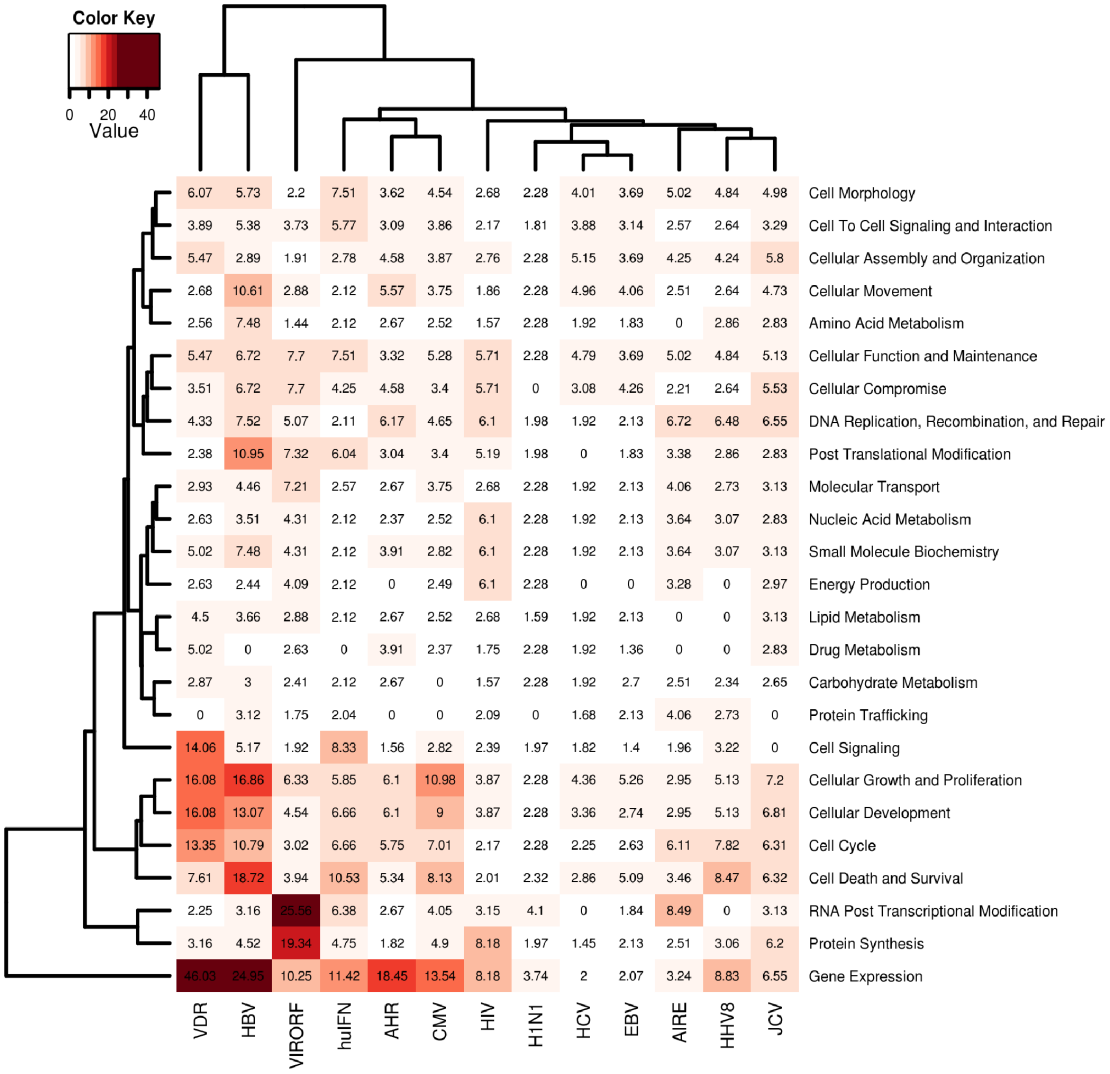
Heatmap from Ingenuity Pathway Analysis of each interactome. Statistical significance (in –log[p-value] notation, where p<0.05 corresponds to a –log[p]>1.3) of the functional components in each interactome, as obtained through a Comparative Core-Analysis in IPA (Ingenuity Pathway Analysis). The functional components identified at the molecular and cellular level are presented row-wise (right); the interactomes are presented column-wise (bottom). Each cell in position (*i,j*) contains a number that represents in −log notation the strength of the association between the functional class *i* and the interactome *j*; this information is also color-matched with a color gradient that moves from white (−log[p] = 0.0, p = 1) to crimson (−log[p] = 50, p<10^−50^). Two hierarchical cluster analyses were employed to group functional classes that share similar patterns of associations across all interactomes (left-side clustering), and to group interactomes that share similar functional compositions (top-chart clustering).

Preliminarily to the enrichment of association analysis, we used IPA to obtain a sense of the cellular signaling pathways that are targeted by each interactome. A classification for molecular and cellular functions showed a comparable distribution of components in most interactomes except for VDR, HBV, VIRORF and hu-IFN where a relative enrichment of some functional pathways (cell signaling, cellular growth and proliferation, cellular development, cell cycle, cell death and survival, protein synthesis, RNA post-transcriptional modification, gene expression) was present (Figure 1).

We investigated statistical enrichment of associations within each one of the above interactomes (Table 1). The analyses were performed with and without considering SNPs falling in the MHC extended region. In both cases the interactomes of EBV, HIV and HBV reached significance. To verify the sensitivity of our results with respect to a choice (SNPs p-value cut-off at 0.05) that is not obvious based on the literature published so far, we evaluated different cut-offs (p<0.005 and p<0.03) on the three interactomes that were MS-associated at p<0.05. These analyses supported the consistency of the results (Table S4).

**Figure 2:**
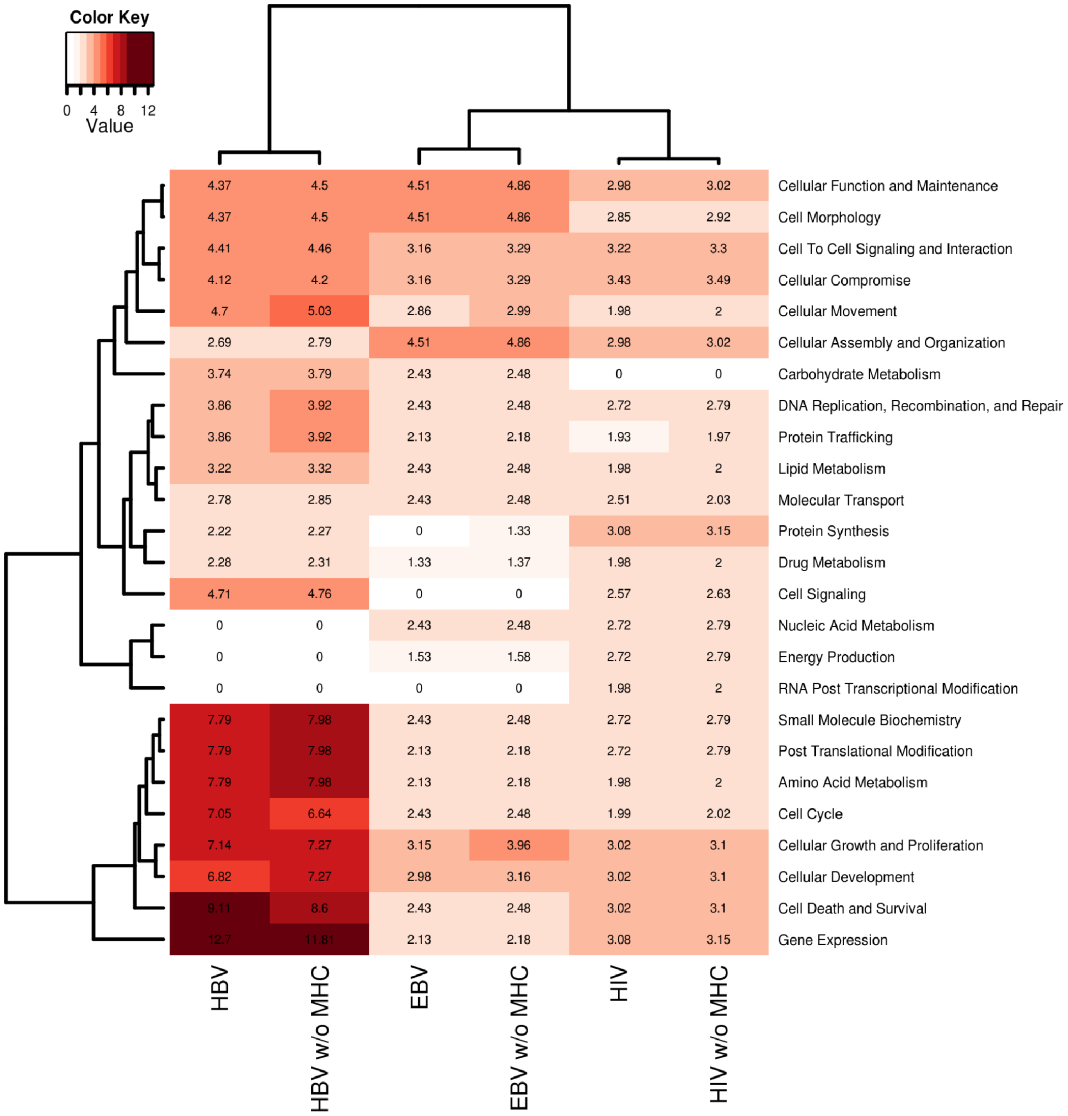
Heatmap from Ingenuity Pathway Analysis of MS-associated interactomes. Statistical significance (in –log[p-value] notation, where p<0.05 corresponds to a –log[p]>1.3) of the functional components in each one of the three MS-associated interactomes (Table S3) computed by ALIGATOR (Association LIst Go AnnoTatOR) first flow process. These p-values were obtained through a Comparative Core-Analysis in IPA (Ingenuity Pathway Analysis). The functional components identified at the molecular and cellular level are presented row-wise; the interactome sub-sets are presented column-wise. Each cell in position (*i,j*) contains a number that represents in −log notation the strength of the association between the functional class *i* and the interactome; this information is also color-matched with a color gradient that moves from white (−log[p] = 0.0, p = 1) to crimson (−log[p] = 14, p<10^−14^). Two hierarchical cluster analyses were employed to group functional classes that share similar patterns of associations across all interactome sub-sets (left-side clustering), and to group interactome sub-sets that share similar functional compositions (top-chart clustering).

**Figure 3:**
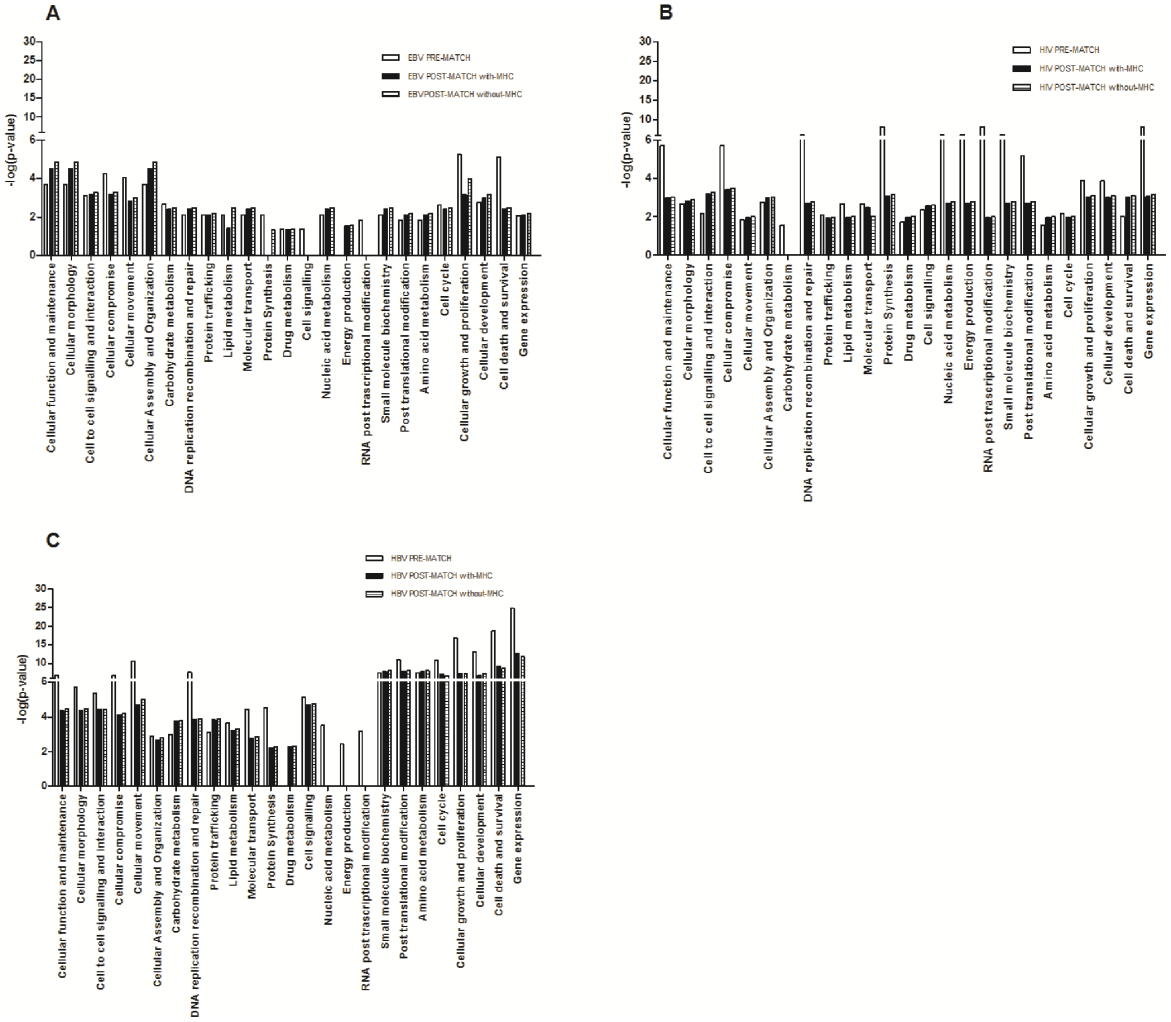
Histograms of functional class distribution of MS-associated interactomes. The histograms show the strength of the association between each IPA functional class and the 3 MS-associated interactomes (EBV [A], HIV [B] and HBV [C]). For each functional class 3 values were derived according to its distribution before (Figure 1) and after (Figure 2, with and without MHC [Major histocompatibility complex]) the ALIGATOR (Association LIst Go AnnoTatOR) statistical analysis of association.

We then performed the same IPA classification as in Figure 1 (Figure 2) on the MS-associated genes within the EBV, HIV and HBV interactomes (Table S3). The aim was to verify whether the associations emerging from the three interactomes implied new and MS-specific perturbations and whether these perturbations are virus-specific or shared by the three pathogens. The comparison between pre- and post-match distribution of the functional classes (Figure 3) showed that the MS-associated interactomes did not reflect a clear cut involvement of specific pathways though, in the case of EBV, an enrichment of some biological functions (cellular function and maintenance, cell morphology, cellular assembly and organization, energy production) was present. On the other hand the most frequent changes for HBV and HIV could be in accord with the post-match reduction of the interactome sizes.

## Discussion

Of the 13 interactomes, 3 show a statistical enrichment of associations. In line with the epidemiological and immunological literature, the EBV interactome is among these. The lack of significant associations with the hu-IFN and AIRE interactomes suggests, though does not exclude, that the result is not an effect of the immunological connotation of the EBV interactome. The absence of associations with the interactomes of phylogenetically related viruses (CMV and HHV8, both herpesviruses with the latter that shares the same site of latency as EBV and belongs to the same subfamily of gamma-herpesviridae) reinforces the specificity of the EBV result. The fact that a portion of the genetic predisposition to MS may be attributable to variants in genes that interact with EBV may be complementary to another our finding showing that EBV genomic variants significantly associate with MS (unpublished data): the two results suggest a model of genetic jigsaw puzzle, whereby both host and virus polymorphisms affect MS susceptibility and, through complex epistatic interactions, eventually lead to disease development.

The associations with the HBV and HIV interactomes were unexpected. Overall, epidemiological data do not support a role of these viruses in the pathogenesis of MS though some controversy still holds concerning the safety of HBV vaccination [19]–[23]. Interestingly, Gregory et al. (2012) [24] demonstrated that in the TNFRSF1A gene, which is part of the HBV interactome, the MS-associated variant directs increased expression of a soluble tumor necrosis factor receptor 1.

Concerning HIV, the lack of epidemiological association seems more established. However, demyelination is a feature of HIV encephalomyelopathy [25] and cases of difficult differential diagnoses or association between the two conditions are described in the literature [26], [27]. All this considered, it might not be surprising that some molecular interactions that take place between HIV and host may predispose to demyelination. Other viruses, sharing homology with HIV may possess better paraphernalia and be more prone to cause MS. The HERV-W family has long been associated with MS [28] and HERV-W/Env, whose expression is associated with MS [29], is able to complement an env-defective HIV strain [30] suggesting a certain degree of functional kinship.

Apart from any conjectures about the data on HBV and HIV interactomes, it remains true, as recently demonstrated by Pichlmair and colleagues (2012) [12], that viruses use unique but also common strategies to perturb the human molecular network. Our pathway analyses do not suggest, in fact, any specific cellular signaling target for the three viruses in MS, perhaps with some exceptions as far as the EBV interactome is concerned. Though preliminary, this acquisition may be in accord with the largely accepted view that, alongside the risk associated with EBV infection, there can be a more general risk of developing MS linked to a variety of other infections [31], [32].

The VDR interactome does not show significant enrichment of associations. The result does by no means diminishes the importance of the epidemiological association between vitamin D and MS: its causal relevance is already supported by data that are starting to explain the molecular basis of this association, upstream [33]–[35], [1] and downstream the interactions between the VDR and its protein cofactors [36], [37].

Current approaches for gene set analysis are in their early stage of development and there are still potential sources of bias or discrepancy among different methods, including those used in our study. As the reproducibility of the techniques increases, and new facilities [38] and methods become available to identify interactions that still escape detection, new lists will become available for matching with GWAS data. In parallel, also the assessment of human genetic variation will become more comprehensive [39]. Hence, the “candidate interactome” approach may become an increasingly meaningful strategy to interpret genetic data in the light of acquisitions from epidemiology and pathophysiology. Notably, this approach appears to be complementary to other studies, which look for statistical enrichment of associations in an unbiased way, and may disclose unexpected pathways in MS susceptibility [40].

At present, our results support a causal role of the interaction between EBV and the products of MS-associated gene variants. Other viruses may be involved, through common and unique mechanisms of molecular perturbation.

## Supporting Information

Table S1 **ALIGATOR settings.**
(XLS)

Table S2 **Composition of all the interactomes.** Lists of genes of each interactome as obtained from the literature. VIRORF =  Virus Open Reading Frame; HIV =  Human Immunodeficiency virus; HCV =  Hepatitis C virus; hu-IFN =  human innate immunity interactome for type I interferon; EBV = Epstein Barr virus; H1N1 =  Influenza A virus; HBV =  Hepatitis B virus; VDR =  vitamin D receptor; AIRE =  autoimmune regulator; CMV =  Cytomegalovirus; HHV8 =  Human Herpesvirus 8; JCV =  JC virus; AHR =  Aryl hydrocarbon receptor.
(DOC)

Table S3 **List of genes within molecular and functional classes in the three MS-associated interactomes (p-value cut-off<0.05).** MS =  multiple sclerosis; HIV =  Human Immunodeficiency virus; EBV =  Epstein Barr virus; HBV =  Hepatitis B virus; MHC =  Major histocompatibility complex
(XLS)

Table S4 **Statistical enrichment of MS-associated interactomes (p-value cut-off<0.005; 0.03).** ALIGATOR-obtained interactome p-values (overall contribution given by SNP p-values to each interactome, with and without SNPs falling in the MHC region). MS =  multiple sclerosis; ALIGATOR = Association LIst Go AnnoTatOR; SNP = single nucleotide polymorphism; MHC = Major histocompatibility complex; HIV =  Human Immunodeficiency virus; EBV = Epstein Barr virus; HBV =  Hepatitis B virus.
(DOC)

## References

[1] International Multiple Sclerosis Genetics Consortium & Wellcome Trust Case Control Consortium,2 (2011) Genetic risk and a primary role for cell-mediated immune mechanisms in multiple sclerosis. Nature 476: 214–19. 21833088 10.1038/nature10251 PMC3182531

[2] Kakalacheva K, Lünemann JD (2011) Environmental triggers of multiple sclerosis. FEBS Lett 585: 3724–29. 21486562 10.1016/j.febslet.2011.04.006

[3] Sawcer S, Wason J (2012) Risk in complex genetics: “All models are wrong but some are useful”. Ann Neurol 72: 502–9. 22605580 10.1002/ana.23613

[4] Visscher PM, Hill WG, Wray NR (2008) Heritability in the genomics era - concepts and misconceptions. Nat Rev Genet 9: 255–266. 18319743 10.1038/nrg2322

[5] Gulbahce N, Yan H, Dricot A, Padi M, Byrdsong D, et al (2012) Viral perturbations of host networks reflect disease etiology. PLoS Comput Biol 8: e1002531. 22761553 10.1371/journal.pcbi.1002531 PMC3386155

[6] Calderwood MA, Venkatesan K, Xing L, Chase MR, Vazquez A, et al (2007) Epstein-Barr virus and virus human protein interaction maps. Proc Natl Acad Sci USA 104: 7606–11. 17446270 10.1073/pnas.0702332104 PMC1863443

[7] Jäger S, Cimermancic P, Gulbahce N, Johnson JR, McGovern KE, et al (2011) Global landscape of HIV-human protein complexes. Nature 481: 365–70. 22190034 10.1038/nature10719 PMC3310911

[8] de Chassey B, Navratil V, Tafforeau L, Hiet MS, Aublin-Gex A, et al (2008) Hepatitis C virus infection protein network. Mol Syst Biol. 4: 230. 10.1038/msb.2008.66 PMC2600670 18985028

[9] Abramson J, Giraud M, Benoist C, Mathis D (2010) AIRE’s partners in the molecular control of immunological tolerance. Cell 140: 123–35. 20085707 10.1016/j.cell.2009.12.030

[10] Li S, Wang L, Berman M, Kong YY, Dorf ME (2011) Mapping a dynamic innate immunity protein interaction. Immunity 35: 426–40. 21903422 10.1016/j.immuni.2011.06.014 PMC3253658

[11] Shapira SD, Gat-Viks I, Shum BO, Dricot A, de Grace MM, et al (2009) A physical and regulatory map of host-influenza interactions reveals pathways in H1N1 infection. Cell. 139: 1255–67. 10.1016/j.cell.2009.12.018 PMC2892837 20064372

[12] Pichlmair A, Kandasamy K, Alvisi G, Mulhern G, Sacco R, et al (2012) Viral immune modulators perturb the human molecular network by common and unique strategies. Nature 487: 486–90. 22810585 10.1038/nature11289

[13] Stark C, Breitkreutz BJ, Chatr-Aryamontri A, Boucher L, Oughtred R, et al (2011) The BioGRID Interaction Database: 2011 update. Nucleic Acids Res. 39: D698–704. 10.1093/nar/gkq1116 PMC3013707 21071413

[14] Holmans P, Green EK, Pahwa JS, Ferreira MA, Purcell SM, et al (2009) Gene ontology analysis of GWA study data sets provides insights into the biology of bipolar disorder. Am J Hum Genet 85: 13–24. 19539887 10.1016/j.ajhg.2009.05.011 PMC2706963

[15] Houlston RS, Cheadle J, Dobbins SE, Tenesa A, Jones AM, et al (2010) Meta-analysis of three genome-wide association studies identifies susceptibility loci for colorectal cancer at 1q41, 3q26.2, 12q13.13 and 20q13.33. Nat Genet 42: 973–77. 20972440 10.1038/ng.670 PMC5098601

[16] Rios D, McLaren WM, Chen Y, Birney E, Stabenau A, et al (2010) A database and API for variation, dense genotyping and resequencing data. BMC Bioinformatics 11: 238. 20459810 10.1186/1471-2105-11-238 PMC2882931

[17] Stajich JE (2007) An Introduction to BioPerl. Methods Mol Biol 406: 535–48. 18287711 10.1007/978-1-59745-535-0_26

[18] Pesole G (2008) What is a gene? An updated operational definition. Gene 417: 1–4. 18457927 10.1016/j.gene.2008.03.010

[19] Confavreux C, Suissa S, Saddler P, Bourdès V, Vukusic S (2001) Vaccines in multiple sclerosis study group. Vaccinations and risk of relapses in multiple sclerosis. N Engl J Med 344: 319–26. 11172162 10.1056/NEJM200102013440501

[20] Ascherio A, Zhang SM, Heman MA, Olek MJ, Coplan PM, et al (2001) Hepatitis B vaccination and the risk of multiple sclerosis N Engl J Med. 344: 327–32. 10.1056/NEJM200102013440502 11172163

[21] Hernán MA, Jick SS, Olek MJ, Jick H (2004) Recombinant hepatitis B vaccine and the risk of multiple sclerosis: a prospective study. Neurology 63: 838–42. 15365133 10.1212/01.wnl.0000138433.61870.82

[22] Mikaeloff Y, Caridade G, Suissa S, Tardieu M (2009) Hepatitis B vaccine and the risk of CNS inflammatory demyelination in childhood. Neurology 72: 873–80. 18843097 10.1212/01.wnl.0000335762.42177.07

[23] Loebermann M, Winkelmann A, Hartung HP, Hengel H, Reisinger EC, et al (2012) Vaccination against infection in patients with multiple sclerosis. Nat Rev Neurol 8: 143–51. 22270022 10.1038/nrneurol.2012.8

[24] Gregory AP, Dendrou CA, Attfield KE, Haghikia A, Xifara DK, et al (2012) TNF receptor 1 genetic risk mirrors outcome of anti-TNF therapy in multiple sclerosis. Nature 488: 508–11. 22801493 10.1038/nature11307 PMC4268493

[25] Johnson RT (1994) The virology of demyelinating diseases. Ann Neurol 36 Suppl: S54–60 10.1002/ana.410360715 PMC7159614 8017889

[26] Berger JR, Sheremata WA, Resnick L, Atherton S, Fletcher MA, et al (1989) Multiple sclerosis-like illness occurring with human immunodeficiency virus infection. Neurology 39: 324–29. 2927638 10.1212/wnl.39.3.324

[27] González-Duarte A, Ramirez C, Pinales R, Sierra-Madero J (2011) Multiple sclerosis typical clinical and MRI findings in a patient with HIV infection. J Neurovirol 17: 504–08. 21965121 10.1007/s13365-011-0054-1

[28] Perron H, Garson JA, Bedin F, Beseme F, Paranhos-Baccala G, et al (1997) Molecular identification of a novel retrovirus repeatedly isolated from patients with multiple sclerosis. The Collaborative Research Group on Multiple Sclerosis. Proc Natl Acad Sci USA 94: 7583–88. 9207135 10.1073/pnas.94.14.7583 PMC23865

[29] Perron H, Germi R, Bernard C, Garcia-Montojo M, Deluen C, et al.. (2012) Human endogenous retrovirus type W envelope expression in blood and brain cells provides new insights into multiple sclerosis disease. Mult Scler Mar 30 [Epub ahead of print]. 10.1177/1352458512441381 PMC3573672 22457345

[30] An DS, Xie YM, Chen IS (2001) Envelope gene of the human endogenous retrovirus HERV-W encodes a functional retrovirus envelope. J Virol 75: 3488–89. 11238877 10.1128/JVI.75.7.3488-3489.2001 PMC114144

[31] Ristori G, Cannoni S, Stazi MA, Vanacore N, Cotichini R, et al (2006) Multiple sclerosis in twins from continental Italy and Sardinia: a nationwide study. Ann Neurol 59: 27–34. 16240370 10.1002/ana.20683

[32] Ascherio A, Munger KL (2007) Environmental risk factors for multiple sclerosis. Part I: the role of infection. Ann Neurol 61: 288–99. 17444504 10.1002/ana.21117

[33] Torkildsen O, Knappskog PM, Nyland HI, Myhr KM (2008) Vitamin D-dependent rickets as a possible risk factor for multiple sclerosis. Arch Neurol 65: 809–11. 18541802 10.1001/archneur.65.6.809

[34] Australia and New Zealand Multiple Sclerosis Genetics Consortium (ANZgene) (2009) Genome-wide association study identifies new multiple sclerosis susceptibility loci on chromosomes 12 and 20. Nat Genet 41: 824–28. 19525955 10.1038/ng.396

[35] Ramagopalan SV, Dyment DA, Calder MZ, Morrison KM, Disanto G, et al (2011) Rare variants in the CYP27B1 gene are associated with multiple sclerosis. Ann Neurol 70: 881–86. 22190362 10.1002/ana.22678

[36] Ramagopalan SV, Heger A, Berlanga AJ, Maugeri NJ, Lincoln MR, et al (2010) A ChIP-seq defined genome-wide map of vitamin D receptor binding: associations with disease and evolution. Genome Res 20: 1352–60. 20736230 10.1101/gr.107920.110 PMC2945184

[37] Disanto G, Sandve GK, Berlanga-Taylor AJ, Ragnedda G, Morahan JM, et al (2012) Vitamin D receptor binding, chromatin states and association with multiple sclerosis. Hum Mol Genet. 21: 3575–86. 10.1093/hmg/dds189 PMC3406756 22595971

[38] Orozco LD, Bennett BJ, Farber CR, Ghazalpour A, Pan C, et al (2012) Unraveling inflammatory responses using systems genetics and gene-environment interactions in macrophages. Cell 151: 658–670. 23101632 10.1016/j.cell.2012.08.043 PMC3513387

[39] Ecker JR, Bickmore WA, Barroso I, Pritchard JK, Gilad Y, et al (2012) Genomics: ENCODE explained. Nature 489: 52–5. 22955614 10.1038/489052a

[40] Baranzini SE, Galwey NW, Wang J, Khankhanian P, Lindberg R, et al (2009) Pathway and network-based analysis of genome-wide association studies in multiple sclerosis. Hum Mol Genet. 18: 2078–90. 10.1093/hmg/ddp120 PMC2678928 19286671

