# Supplementary material for: A “Candidate-Interactome” Aggregate Analysis of Genome-Wide Association Data in Multiple Sclerosis
Source: PLoS One. 2013 May 16;8(5):e63300. doi: 10.1371/journal.pone.0063300 (PMC3655974; doi:10.1371/journal.pone.0063300)
Supplement: Table S2 — Composition of all the interactomes. Lists of genes of each interactome as obtained from the literature. VIRORF = Virus Open Reading Frame; HIV = Human Immunodeficiency virus; HCV = Hepatitis C virus; hu-IFN = human innate immunity interactome for type I interferon; EBV = Epstein Barr virus; H1N1 = Influenza A virus; HBV = Hepatitis B virus; VDR = vitamin D receptor; AIRE = autoimmune regulator; CMV = Cytomegalovirus; HHV8 = Human Herpesvirus 8; JCV = JC virus; AHR = Aryl hydrocarbon receptor. (DOC) [file pone.0063300.s002.doc]

Table S2: Composition of all the interactomes

| **VIRORF** |
| --- |
| ACACA; ACAD11; ACAP2; ACLY; ACOT8; ACP1; ACTA2; ACTBL2; ACTG2; ADAR; AHCY; AIFM1; ALDOA; ANP32A; ANP32B; ANXA1; ATAD3A; ATP1A1; ATP1A3; ATP5A1; ATP5B; ATP5C1; ATP6AP1; ATP6AP2; ATXN2L; BAG2; BASP1; BAZ1B; BEND4; BIN1; BLOC1S1; C10orf32; C10orf90; C14orf166; C22orf28; CALM1; CALML5; CALR; CASP8; CBX3; CCT2; CCT3; CCT4; CCT5; CCT6A; CCT7; CCT8; CDC37; CEP55; CFL1; CGGBP1; CHUK; CKB; CLPX; CLTC; COBLL1; COPB1; CPSF1; CPSF6; CREBBP; CSDA; CSNK2A1; CSNK2B; CTBP1; CTBP2; CTSG; CUL3; CUL4A; CUL7; CUL9; CYB5R4; DARS2; DDA1; DDB1; DDX17; DDX19A; DDX19B; DDX21; DDX28; DDX39B; DDX3X; DDX3Y; DDX4; DDX5; DDX6; DECR1; DEFA1; DEFA3; DEFA4; DHX15; DHX30; DHX9; DLG5; DMBT1; DNAJA1; DNAJA2; DNAJA3; DNAJB11; DSP; DYNC2H1; DYNLL1; DYNLL2; EDC4; EEF1A1; EEF1A1P5; EEF1A2; EEF2; EIF2B1; EIF2B2; EIF2B3; EIF2B4; EIF2B5; EIF2S3; EIF2S3L; EIF4H; EIF5A; EIF5A2; EIF5AL1; ELANE; ELAVL1; ENO1; ENO2; ENO3; ENY2; EP300; ERH; FAM125A; FAM125B; FAM98B; FASN; FAU; FBL; FBXO3; FBXW11; FFR; FKBP8; FLNB; FUS; GAPDH; GART; GDI2; GNB1; GNB2; GNB2L1; GNB3; GNB4; GOLGA2; GRPEL1; GTF2B; GTF2I; H1FX; H2AFJ; H2BFS; H3F3A; H3F3C; HADHB; HAX1; HBD; HDAC1; HDAC2; HIST1H1A; HIST1H1T; HIST1H2AB; HIST1H2AC; HIST1H2AD; HIST1H2AG; HIST1H2AH; HIST1H2AJ; HIST1H2BA; HIST1H2BB; HIST1H2BC; HIST1H2BD; HIST1H2BH; HIST1H2BJ; HIST1H2BJ; HIST1H2BK; HIST1H2BL; HIST1H2BM; HIST1H2BN; HIST1H2BO; HIST1H3A; HIST1H4A; HIST2H2AA3; HIST2H2AC; HIST2H2BE; HIST2H2BF; HIST2H3A; HIST3H2A; HIST3H2BB; HIST3H3; HNRNPA0; HNRNPA1; HNRNPA1L2; HNRNPA2B1; HNRNPA3; HNRNPAB; HNRNPC; HNRNPD; HNRNPF; HNRNPH1; HNRNPH2; HNRNPH3; HNRNPK; HNRNPL; NRNPR; HNRNPU; HNRNPUL1; HNRPDL; HRNR; HSD17B10; HSP90AA1; HSP90AB1; HSP90B1; HSPA1L; HSPA2; HSPA4; HSPA4L; HSPA5; HSPA6; HSPA7; HSPD1; HSPE1; HSPH1; HYOU1; IFIH1; IGF2BP1; IGF2BP2; IGF2BP3; IGHA1; IGHG1; IGHG2; IGKC; IGLC1; IGLC2; IGLC3; IGLC6; IKBKB; IKBKG; ILF2; ILF3; IRF3; IRS4; ISYNA1; JUP; KCTD9; KDM1A; KEAP1; KHDRBS1; KHNYN; KHSRP; KIAA0913; KIAA1967; KIF3C; KIF5B; KLHL7; KNDC1; KPNA1; KPNA3; KPNA4; KPNA5; KPNA6; KPNB1; LARP1; LDHA; LDHB; LEMD2; LENG8; LGALS3BP; LOH12CR1; LTF; LYZ; MACC1; MAD1L1; MAN1B1; MAP4K4; MARCKS; MATR3; MCCC1; MCCC2; MCM2; MDH2; MIB2; MIF; MOBKL1A; MOBKL1B; MOV10; MPO; MRPL12; MRPS27; MTA1; MTA2; MYBBP1A; MYH10; MYH14; MYH9; MYL6; MYL6B; MYO1C; MYO5A; MYO5C; MYO9A; NAP1L1; NAP1L4; NBPF8; NCL; NEFH; NHS; NISCH; NKRF; NME4; NPM1; NUDT21; NUP153; NUP98; NYNRIN; OCIAD2; OCRL; PABPC1; PABPC4; PABPN1; PAICS; PCBP1; PCBP2; PCBP3; PCCA; PCCB; PCID2; PCMT1; PFKP; PFN1; PGAM1; PGAM2; PGAM4; PGAM5; PGK1; PHB2; PHLDB2; PHLDB3; PIK3CA; PIK3CB; PIK3R1; PIK3R2; PIK3R3; PIP; PLP2; POLD1; POLDIP2; POLR2A; POLR2B; POM121; POM121C; POTEE; POTEF; POTEI; POTEJ; PPAN; PPIA; PPP1CA; PPP1CB; PPP1CC; PPP2CA; PPP2R1A; PPP2R2A; PPP2R5A; PPP2R5C; PPP2R5D; PPP2R5E; PRDX1; PRDX2; PRDX3; PRDX4; PRDX6; PRKCSH; PRKRA; PRTN3; PSIP1; PSMD12; PSPC1; PTBP1; PTMA; R13AX_HUMAN; RA1L3_HUMAN; RAB10; RAE1; RALY; RBBP4; RBBP7; RBM14; RBMX; RILPL1; ROCK2; RPL10A; RPL11; RPL12; RPL13A; RPL14; RPL15; RPL17; RPL18; RPL18A; RPL19; RPL21; RPL22; RPL23; RPL23A; RPL24; RPL26; RPL26L1; RPL27; RPL27A; RPL28; RPL3; RPL30; RPL31; RPL32; RPL34; RPL35; RPL35A; RPL36; RPL36A; RPL36AL; RPL37A; RPL38; RPL4; RPL5; RPL6; RPL7; RPL7A; RPL8; RPL9; RPLP0; RPLP0P6; RPLP1; RPS10; RPS11; RPS12; RPS13; RPS15A; RPS16; RPS17; RPS18; RPS19; RPS2; RPS20; RPS21; RPS23; RPS24; RPS26; RPS27; RPS27L; RPS3; RPS3A; RPS4X; RPS5; RPS6; RPS7; RPS8; RPS9; RPSA; RUVBL1; RUVBL2; S100A7; SARNP; SEC24C; SEC24C; SEC31A; SERBP1; SERPINA12; SF3B2; SF3B3; SFPQ; SLC25A1; SLC25A3; SLC25A31; SLC25A4; SLC25A5; SLC25A6; SMARCA4; SMARCC2; SMARCE1; SMYD3; SNRPA; SNRPD3; SNRPF; SOLH; SPAG17; SPATA7; SPIRE1; SPOP; SPRR1A; SPRR1B; SPRR2A; SPRR2B; SPRR2D; SPRR2E; SPRR2G; SPTBN1; SPTBN2; SRP9; SRSF4; SRSF5; SRSF6; SSB; STAT1; STAT2; STAU1; STMN1; STUB1; SUMO2; SYNCRIP; SYNE1; TAF15; TBB8B_HUMAN; TCEB1; TCEB2; TCP1; TFG; THOC4; TIMM50; TMEM109; TP53; TPI1; TRAF6; TRIM21; TRIM28; TRIM56; TSG101; TSGA10; TSSC1; TUBA1A; TUBA1C; TUBA3C; TUBA3E; TUBA4A; TUBB; TUBB2A; TUBB2B; TUBB2C; TUBB3; TUBB4; TUBB6; TUBB8; TUFM; TXN; UBR5; UNG; UPF1; USP19; USP24; USP54; USP7; USP9X; VAPA; VAPB; VAT1; VCP; VMA21; VPS26B; VPS28; VPS35; VPS37B; VPS52; VPS53; VPS54; WDR6; WNK1; WNK2; WNK3; WRNIP1; XPO5; XRN2; YBX1; YBX1; YI028_HUMAN; YWHAB; YWHAE; YWHAG; YWHAQ; YWHAZ; ZMYND11; ZNF346; ZNF516 |
| **HIV** |
| ABCB10; ABCD3; ACBD5; ACOT8; ACSL3; AFF1; AFF4; AGPS; AHCY; AHCYL1; AHCYL2; AIMP1; AIMP2; AKAP8L; AKAP9; ALMS1; AMBRA1; ANAPC1; ANAPC2; ANKHD1; ANKRD13A; ANKRD17; AP1M1; APP; ARFGEF1; ARFGEF2; ARHGEF12; ARRB2; ATAD3A; ATAD3B; ATL3; ATP2A2; ATP2A3; ATP2B1; ATP5B; ATP5D; ATP5F1; ATP5H; ATP5J; ATP5O; ATXN10; BAG6; BCAP31; BICD2; BTRC; C20orf24; CALR; CALU; CAND2; CANX; CAPN1; CARM1; CASC3; CBFB; CCDC47; CCNT1; CDK9; CDKN2A; CEP170; CEP57; CERS2; CHAF1B; CHCHD1; CHI3L2; CHP; CIAO1; CKAP4; CKMT1A; CKMT1B; CLGN; CLN6; CNTNAP1; COMT; COPS2; COPS3; COPS4; COPS5; COPS6; COPS8; COX4I1; COX5A; COX6B1; CSDE1; CTNNA3; CTSB; CTSD; CUL1; CUL2; CUL4A; CUL5; CYB5R1; DAGLB; DARS; DCAF11; DCAF16; DCAF8; DDA1; DDB1; DDX20; DDX49; DDX6; DHCR24; DIAPH1; DNAJB11; DNAJB2; DNAJC3; DNAJC7; DOHH; DOK1; DPY19L1; DPY19L3; DSC3; DSP; DYNC1H1; DYNC1I2; DYNC1LI1; EDC4; EDEM3; EEF1E1; EFEMP1; EIF2AK2; EIF3A; EIF3B; EIF3C; EIF3D; EIF3E; EIF3F; EIF3G; EIF3H; EIF3I; EIF3K; EIF3L; EIF3M; EML3; ENDOD1; EPPK1; EPRS; ERGIC1; ERGIC2; ERGIC3; ERLEC1; ERP44; EVL; EXOC4; FAM133B; FAM195A; FAM203A; FAM96B; FAM98A; FBN2; FBXO3; FBXO6; FBXW11; FECH; FKBP4; FLAD1; FLCN; FLOT1; FNIP1; FOXC1; FUT8; G3BP1; G3BP2; GAK; GANAB; GEMIN2; GEMIN4; GEMIN6; GEMIN7; GIMAP5; GLG1; GLT25D1; GOLGA1; GOLPH3; GPATCH4; GPS1; GPS2; GRB2; HADHA; HADHB; HARS2; HAT1; HDAC3; HDDC2; HEATR1; HERC2; HIST1H3A;  HIST1H3B; HIST1H3C; HIST1H3D; HIST1H3E; HIST1H3F; HIST1H3G; HIST1H3H; HIST1H3I;  HIST1H3J; HLA-A; HLA-B; HLA-C; HM13; HNRNPH3; HSD17B12; HSP90B1; HSPA13; HSPA6; HSPA7; HSPH1; HUWE1; HYOU1; IARS; ICAM2; IDH3A; IFT122; IK; IPO4; IPO9; ITGA4; ITGAL; ITGB2; ITM2B; IVNS1ABP; KARS; KIAA0090; KIAA1429; KIAA1967; KIAA2013; KIF21B; KIR3DL1; KIR3DL2; LARP7; LARS; LDLR; LGALS3BP; LIMD1; LMAN1; LMAN2; LRPPRC; LRRC47; LSM12; MAGED1; MAGT1; MAPK6; MARS; MATR3; MCL1; MCM4; MCM6; MCM7; MEPCE; MESDC2; MITD1; MLLT1; MMGT1; MOGS; MPG; MRPL11; MRPL42; MRPS28; MRPS9; MRS2; MSH2; MSN; MTCH1; MTCH2; MTHFD2; NAP1L4; NARS2; NCAPD3; NCOR1; NDUFB10; NDUFS8; NEDD4L; NEDD8; NFAT5; NFU1; NGLY1; NHP2L1; NMT1; NOL12; NOLC1; NPM3; NUDC; OLA1; OS9; OSBPL3; OSBPL6; P4HA2; P4HB; PARP4; PDIA3; PDIA4; PDIA5; PDIA6; PELO; PGRMC1; PHB; PHB2; PIP4K2A; PLAA; PLOD1; PLOD2; PLSCR1; POLE; POLE3; POLE4; PON2; PPM1G; PPP1R12A; PPP2CB; PPP2R5E; PRDX4; PREB; PRKCSH; PRKDC; PRMT1; PSIP1; PSMA1; PSMB6; PSMD11; PSMD7; PSME3; PTGES3; PTPLAD1; PUM2; QARS; RABGAP1; RANBP1; RANBP2; RANBP6; RARS; RBBP7; RBM17; RCN1; RCN2; RECQL; RER1; RHOC; RNF126; RNF213; RNF5; RNF7; RNH1; RPN1 RRM2; RTN3; RTN4; RUVBL1; SARS; SCAMP3; SDCBP; SDCCAG8; SDF2; SDF2L1; SDF4; SDHA; SEC61A1; SEC62; SEH1L; SEL1L; SEPSECS; SERPINH1; SF3A3; SGPL1; SIRT1; SKP1; SLC27A4; SLC30A7; SLC9A3R1; SLIRP; SMG6; SMN1; SMN2; SMU1; SPAG5; SPATS2; SPCS2; SPCS3; SQSTM1; SRRM2; SSBP1; SSR4; SSSCA1; STOML2; STRAP; STUB1; SUCLA2; SUMF2; SUN1; SURF4; TAB2; TARS; TBC1D15; TBL1XR1; TBRG4; TCEB1; TCEB2; THRAP3; THUMPD1; TIMM13; TIMM8A; TIMM8B; TMED10; TMED4; TMED9; TMEM11; TMEM160; TMEM192, TMEM38B; TMEM43; TMEM55B; TOMM40; TOR1A; TOR1AIP2; TRBV12-3; TRPS1; TULP3; TXNDC5; TYSND1; UBA1; UBE2O; UBL4A; UBR2; UBR4; UGGT1; UNC45A; UNG; USP11; VAPA; VBP1; VCP; VDAC1; VDAC2; VDAC3; VPRBP; VTI1A; WDR61; XPO1; XPO4; XPO5; YTHDF3; YWHAB; YWHAE; YWHAG; YWHAZ; ZFP91; ZNF579 |
| **HCV** |
| ACTN1; ACTN2; AEBP1; AGRN; ANKRD12; ANKRD28; ARFIP2; ARHGEF6; ARNT; ARS2; ASXL1; BCAN; BCAR; BCL6; BIN1; BZRAP1; C10orf18;,C10orf6; C12orf41; C1orf165; C1orf94; C7; C9orf30; CALCOCO2; CBY1; CCDC37; CCDC52; CCDC66; CCDC95; CCHCR1; CD5L; CD68; CDC23; CELSR2; CEP152; CEP192; CEP68; CFP; CHPF; COL4A2; CORO1B; CSNK2B; CTGF; CXorf45; DEAF1; DES; DPF1; DPP7; EFEMP1; EFEMP2; EIF4ENIF1; ELAC2; FAM120B; FBLN1; FBLN2; FBLN5; FBN1; FBN3; FES; FIGNL1; FLAD1; FLJ11286; FN1; FRMPD4; FRS3; FTH1; FUCA2; FXYD6; GAA; GAPDH; GFAP; GNB2; GON4L; GRN; HIVEP2; HOMER3; HOXD8; HSPA5; IQWD1; ITGB1; ITGB4; JAG2; JUN; JUN; KHDRBS1; KIAA1411; KIAA1549; KIF17; KIF7; L3MBTL3; LAMA5; LAMB2; LAMC3; LDB1; LMNB1; LOC728302; LOC730765; LPXN; LRRCC1; LRRTM1; LTBP4; LZTS2; MAGED1; MAPK7; MEGF6; MEGF8; MGC2752; MLLT4; MLXIP; MMRN2; MORC4; MVP; N-PAC; NAP1L1; NCAN; NEFL; NEFM; NELL1; NELL2; NID1; NID2; NOTCH1; NR4A1; NUP62; OBSCN; PAK4; PARP4; PCYT2; PDE4DIP; PDLIM5; PFN1; PICK1; PKNOX1; PLEKHG4; PLSCR1; PNPLA8; POU3F2; PSMA6; PSME3; PTPRN2; RABEP1; RAI14; RBM4; RCN3; RGNEF; RICS; RINT1; RNF31; RSHL2; RUSC2; SBF1; SDCCAG8; SECISBP2; SEPT10; SERTAD1; SESTD1; SETD2; SF3B2; SIAH1; SLC31A2; SLIT1; SLIT2; SLIT3; SMEK2; SMYD3; SNX4; SPOCK3; SPON1; SRPX2; SSX2IP; STAB1; STAT3; SVEP1; TAF1; TBC1D2B; TGFB1I1; THAP1; TMSB4X; TRIM23; TRIM27; TRIO; TRIP11; TUBB2C; TXNDC11; UBQLN1; UBQLN4; USHBP1; USP19; UXT; VCAN; VIM; VWF; XAB2; XRN2; YY1AP1; ZBTB1; ZCCHC7; ZHX3; ZNF271; ZNF281; ZNF410; ZZZ3 |
| **hu-IFN** |
| AIP; APBA3; ARAF; ATG7; ATG9A; AZI2; BAG6; BIRC2; CALCOCO2; CCDC47; CDC37; CDC42BPG; CHUK; CLPTM1; COG7; COPS2; CREBBP; CSTF1; CTBP1; CTBP2; DDOST; DDX58; DHX30; DHX9; DIABLO; DICER1; DZIP3; EDC4; EIF2AK2; EIF2C2; EIF6; EP300; FOXK1; FOXK2; FTSJ3; GHITM; IFRD2; IKBKAP; IKBKB; IKBKE; IKBKG; IRAK1; IRAK2; IRAK4; IRF2BP1; IRF2BP2; IRF3; IRF6; IRF8; KEAP1; KIAA1274; LTN1; MAP3K7; MAVS; MBOAT7; MIB1; MOV10; MPP1; MPP6; MTPAP; N4BP1; NACA; NKRF; NLRX1; OAS3; OPTN; PEG10; PLXDC2; PPP3CB; PRKRA; PYGL; RB1; RB1CC1; RBCK1; RBL1; RELA; RIPK1; RIPK2; RNF31; SAMHD1; SGPL1; STAT1; STAT2; STAU2; STT3A; SURF4; TAB1; TAB2; TAB3; TANK; TARBP2; TARDBP; TBK1; TBKBP1; TLK2; TLR4; TOLLIP; TOM1L1; TRAF1; TRAF2; TRAF3; TRAF6; TRAF7; TRIM32; TRMT61B; TXLNA; TXLNB; UBE4A; UNC93B1; WRNIP1; YTHDC2; ZC3H12A; ZNF346 |
| **EBV** |
| ACTN1; ACTN4; AES; AP2B1; APOL3; ARHGEF10L; BAG6; BLNK; C1QBP; CASKIN2; CBX3; CCDC14; CCHCR1; CD46; CD5L; CD74; CERS4; CIR1; CLK1; CTSC; DKK3; DYNLL1; EFEMP1; EFEMP2; EGFL7; ELF2; ENOX2; FAM168B; FBLN2; FBLN5; FCHSD1; FN1; GAPDH; GCA; GOLGA6L5; GPRASP1; GPRASP2; GRN; HBB; HLA-A; HLA-B; HOMER3; IGHG1; IGLL1; IMMT; IQGAP2; KPNA2; KRT36; LAMB1; LAMB2; LGALS3BP; LMNB1; LTBP4; LZTS2; MAPK7; MAPRE1; MARCO; MDFI; MFSD1; MMRN2; NCKIPSD; NFKB1; NR4A1; NTN4; NUCB1; NUCB2; OPTN; PARP4; PDE6G; PHYHD1; PICK1; PIGS; PKM2; PLSCR1; PPP1CA; PSME3; RBCK1; RBP1; RBPMS; RCBTB1; RNF31; RPL3; RPL4; SEPP1; SERTAD1; SH3GLB1, SHROOM3; SLIT2; SLIT3; SNX4; SP100; SRI; TES; TMEM66; TNXB; TRA2B; TRAF1; TRAF2; TRAF3IP3; TSC22D4; TSG101; TSNARE1; TUBA1A; TXNDC11; UBE2I; UBXN11; VIM; VWF; ZMYND11; ZYX |
| **H1N1** |
| ABLIM1; ACOT9; AIMP2; BANP; BCAP29; BHLHE40; BLZF1; C10orf35; C10orf96; C16orf45; C1orf94; CALCOCO1; CBS; CCDC33; CDC42EP4; CEP70; CHMP1B; CHMP6; CMTM5; COL4A3BP; CREB3; CRYAB; DOCK8; DVL2; DVL3; DYNLL2; EXOSC8; FXR2; GABPB1; GLYAT; GMCL1; HOOK1; IKZF3; KCNRG; KHDRBS3; KIAA1143; KPNA3; KPNA6; LINC00525; LNX2; LYPLA1; MAGEA11; MAGEA6; MAGED1; MAPK9; MEOX2; MIPOL1; MLH1; MTAP; NBPF22P; NCAPH2; NDUFS3; NRF1; PLAC8; PNMA1; PPP2R5C; PRKRA; QTRT1; RABGEF1; RBPMS; RNF5; SDCBP2; SECISBP2; SEPT1; SETBP1; SIAH1; SP100; SSBP2; STAU1; STX5; TACC1; TARBP2; TCF12; TFCP2; TMEM86B; TRAF1; TRAF2; TRIP6; TTC12; UBE2I; UROS; USHBP1; YIPF6; ZBTB25; ZMAT3; ZMAT4; ZNF346 |
| **HBV** |
| AIP; AKT1; ALB; AR; ASGR1; ATF2; ATF3; BAX; BHMT2; CAK; CALR; CCR5; CD44; CD81; CDC2; CFLAR; CREB1; CREM; DBI; DDB1; DDB2; DNAJA3; DNAJB1; DNMT3A; E2F1; EEF2; EGR1; ERCC2; ERCC3; FETUB; FGB; FOXM1; GTF2B; GTF2H2; GTF2H3; GTF2H4; GTF2H5; GTF3A; HBX1P; HDAC1; HNF4A; HSP90AA1; HSP90B1; HSPA1A; HSPA5; ID1; IL6; JAG1; JAK1; JUN; MAP2K4; MAPK1; MAPK8; MAPK9; MAZ; MEKK1; MIF; MPDU1; MRPL41; MT2A; NFKB1; NFKBIA; NFKBIE; P53; PIK3CA; PKC; POLG; POLR2A; POLR2E; PPP2CA; PSMA7; PSMC1; PTPN3; SKP2; SLC25A23; SMAD4 ; SREBF1; SRPK1; SRPK2; STAT1; TBP; TM4SF4; TPSB2; UGT1A9; VBP1 |
| **VDR** |
| ACTN4; BAG1; BAZ1B; CASC4; CDK11B; CDKN1A; CEBPA; CLASRP; COPS2; CREBBP; EIF3I; EP300; FOS; FOXO3; FOXO4; GABARAPL1; GABARAPL2; GNPAT; GRIP1; HDAC2; HMGN3; HNF4A; HR; JUN; KDM5A; LCOR; MAPK1; MAPK3; MED1; MED12; MED13; MED14; MED16; MED21; MED23; MED24; MED4; MED6; MED7; NCOA1; NCOA2; NCOA3; NCOA4; NCOA6; NCOR1; NCOR2; NR0B2; NR1H2; NR2E3; NRIP1; POLR2A; POU1F1; POU2F1; PPP1CC; PPP1CC; RB1; RNF14; RUNX1; RUNX1T1; RXRA; RXRB; RXRG; SIN3A; SIRT1; SMAD3; SMARCA4; SNW1; SRC; STAT1; TAF11; TAF7; TCF3; TDG; THRAP3; TP53; TRRAP; UBC; VDR; ZBTB16 |
| **AIRE** |
| C1QBP; CAND1; CHD6; DDX17; DDX5; EFTUD2; GCN1L1; GEMIN5; HIST1H2AC; HIST1H2BO; HIST3H3; IPO7; KPNB1; LMNB1; MCM2; MCM5; MCM6; MSH2; MSH6; MYBBP1A; NASP; NOP56; NUP93; PABPC1; PARP1; PCNA; POLR2A; POLR2B; PRKDC; RAD21; RANBP2; RANBP9; RUVBL2; SMC1A; SMC3; SNRPB; SNRPD3; SRSF1; SRSF2; SRSF3; SUPT16H; TOP2A; TRIM28; XPO1; XPOT |
| **CMV** |
| ANXA2; B2M; BAX; CD209; CREB1; DAXX; DR1; EGFR; EGR1; GTF2B; HDAC1; HDAC2; HDAC3; HNRNPA1; JUN; JUNB; KAT2B; KPNA1; KPNA3; KPNA4; LILRB1; MICA; MTA2; NFE2; NFE2L2; RAB11FIP4; RB1; SPI1; STAT1; STAT2; SUMO2; SUPT6H; TAP1; TAP2; TAPBP; TBP; TEAD1; TP53; TSC1; TSC2; UBE2I |
| **HHV8** |
| ASF1B; BRD4; CA4; CDK6; CDKN1B; CEBPA; CHEK1; CHUK; COPS5; DEK; EIF3M; ERCC2; GFPT2; GSK3B; HIST1H2AG; HIST1H2AI; HIST1H2AK; HIST1H2AL; HIST1H2AM; HIST1H2BC; HIST1H2BE; HIST1H2BF; HIST1H2BG; HIST1H2BI; IKBKG; JUN; MECP2; MED15; MED16; MED24; PCNA; POLD2; POU2F1; RB1; RBPJ; RRM1; SDF2; STAT3; STAT5A; SUN2 |
| **JCV** |
| CTNNB1; FOS; HSPA8; IRS1; JUN; PPP2R4; PURA; RB1; TP53; YBX1 |
| **AHR** |
| AIP; ARNT; ARNTL; BRCA1; CCNT1; CTNNB1; DAP3; DDB1; EP300; ESR1; FOXA1; GTF2F1; GTF2F2; HSP90AA1; IVNS1ABP; NCOA2; NCOA7; NCOR2; NEDD8; NR2F1; NRIP1; RB1; RELA; SMARCA4; SP1; SRC; STUB1; SUMO2; TBP; UBC |

Lists of genes of each interactome as obtained from the literature.

VIRORF= Virus Open Reading Frame; HIV= **Human Immunodeficiency virus;** HCV= Hepatitis C virus; hu-IFN= human innate immunity interactome for type I interferon; EBV=Epstein Barr virus; H1N1= Influenza A virus; HBV= Hepatitis B virus; VDR= vitamin D receptor; AIRE= autoimmune regulator; CMV= Cytomegalovirus; HHV8= Human Herpesvirus 8; JCV= JC virus; AHR= Aryl hydrocarbon receptor.
