# Supplementary material for: A “Candidate-Interactome” Aggregate Analysis of Genome-Wide Association Data in Multiple Sclerosis
Source: PLoS One. 2013 May 16;8(5):e63300. doi: 10.1371/journal.pone.0063300 (PMC3655974; doi:10.1371/journal.pone.0063300)
Supplement: Table S4 — Statistical enrichment of MS-associated interactomes (p-value cut-off<0.005; 0.03). ALIGATOR-obtained interactome p-values (overall contribution given by SNP p-values to each interactome, with and without SNPs falling in the MHC region). MS = multiple sclerosis; ALIGATOR = Association LIst Go AnnoTatOR; SNP = single nucleotide polymorphism; MHC = Major histocompatibility complex; HIV = Human Immunodeficiency virus; EBV = Epstein Barr virus; HBV = Hepatitis B virus. (DOC) [file pone.0063300.s004.doc]

**Table S4:** **Statistical enrichment of MS-associated interactomes (p-value cut-off<0.005; 0.03).**

|  | 0.005 | | 0.03 | |
| --- | --- | --- | --- | --- |
|  | p-value with MHC | p-value without MHC | p-value with MHC | p-value without MHC |
| EBV | 0,0044 | 0,0326 | 0,0008 | 0,0054 |
| HBV | 0,0208 | 0,0464 | 0,0016 | 0,0084 |
| HIV | 0,0001 | 0,0002 | 0,0058 | 0,0082 |

ALIGATOR-obtained interactome p-values (overall contribution given by SNP p-values to each interactome, with and without SNPs falling in the MHC region).

MS= multiple sclerosis; ALIGATOR = Association LIst Go AnnoTatOR; SNP = single nucleotide polymorphism; MHC = Major histocompatibility complex; HIV= **Human Immunodeficiency virus;** EBV=Epstein Barr virus; HBV= Hepatitis B virus.
